# Assessment of the effects of bean extract on axillary hair reduction

**DOI:** 10.22038/AJP.2023.22888

**Published:** 2024

**Authors:** Fereshteh Zamiri, Hassan Rakhshandeh, Bita Kiafar, Syed Mohammad Naqvi, Maryam Emadzadeh, Sara Fakhraei, Masoud Maleki

**Affiliations:** 1 *Department of Dermatology, Faculty of Medicine, Mashhad University of Medical Sciences, Mashhad, Iran*; 2 *Pharmacological Research Center of Medicinal Plants, Mashhad University of Medical Sciences, Mashhad, Iran*; 3 *Cutaneous Leishmaniasis Research Center, Mashhad University of Medical Sciences, Mashhad, Iran*; 4 *Student Research Committee, Mashhad University of Medical Sciences, Mashhad, Iran *; 5 *Clinical Research Development Unit, Ghaem Hospital, Mashhad University of Medical Sciences, Mashhad, Iran*

**Keywords:** Axillary hairs, Trichoscopy, Broad bean, Hair reduction

## Abstract

**Objective:**

Body hair removal plays an important role in beauty standards, particularly for women. Finding a method that is easy to use, cheap, and can be done without supervision can significantly affect long-term hair reduction and reduce the side effects of hair removal. The present study investigated the impact of a containing 20% broad bean (*Vicia faba*) extract cream on axillary hair removal.

**Materials and Methods:**

Twenty-five female volunteers were randomly divided into A (right axillary intervention - left axillary placebo) and B (right axillary placebo - left axillary intervention). Depending on the group, each person used a cream containing 20% broad bean extract )"The extract made from the seeds and pods of broad beans.") on one side and a placebo on the other twice a day for three months. Volunteers shaved their axillary hairs three days before each visit and took pictures of both sides on the day of the visit with a trichoscope (to check the diameter and thickness of the hairs).

**Results:**

We found a decrease in thickness on the intervention group (the axilla where a cream containing broad bean extract was applied); however, this difference was not significant between the intervention side and the placebo. In terms of the number of hairs, the difference between the two groups was significant only in the second month despite the decrease on the intervention side. Evaluation based on the personal judgment of the volunteers showed that there was a substantial difference in terms of the number of hairs (p=0.012) and thinning of hair (p=0.02).

**Conclusion:**

Our findings showed that 20% broad bean extract cream could potentially reduce axillary hair growth.

## Introduction

All mammalian body surfaces are covered with hair except for small areas such as the palms, soles, buccal surface of the lips, and parts of the external genitalia. Body hair can be divided into androgen-dependent hair, such as the scalp, beard, chest, axilla, and pubic hair, and androgen-independent such as the eyebrows, eyelashes, and vulvar hair (Buffoli et al., 2014). Excess facial hair is found in about 41 million women in the United States (Hamzavi et al., 2007).

There is a growing trend of feeling the need to remove body hair for cosmetic reasons, particularly among women; furthermore, disorders such as hypertrichosis and hirsutism, in addition to cosmetic problems, can cause psychological dysfunctions such as anxiety and depression (Clatici et al., 2020). This need has led to various methods being used to remove body hair. Some of these methods, such as shaving, waxing, and using depilatories, are temporary and need to be used frequently but can be associated with side effects such as skin irritation and allergic reactions (Wanitphakdeedecha et al., 2012). Other methods, such as electrolysis and laser hair removal, while being longer term solutions are also more expensive and require an experienced operator to be performed. Moreover, if not done correctly or the client is not selected properly, it can be associated with complications such as burns, pigmentation changes, scars, reactivation of herpes infection, and paradoxical hypertrichosis (Clatici et al., 2020; Hamzavi et al., 2007). So far, various methods such as Eflornitin cream approved by the US Food and Drug Administration, have been used to reduce facial hairs in idiopathic hirsutism (Hamzavi et al., 2007). Compounds such as finasteride gel (Tahvilian et al., 2015), and fennel 1% and 3% gel (Akha et al., 2014; Javidnia et al., 2003), have also been used in small studies and have been somewhat effective. Curcuma aeruginosa roxb oil has also been used for axillary hair removal in one study and it could reduce hair growth to some extent (Srivilai et al., 2017).

In the present study, we investigated the effect of a cream containing broad bean extract on axillary hair. The Faba bean, also called broad bean or *Vicia faba,* is an important member of the legume family (Ray and Georges, 2010). This plant contains nutritional factors such as carbohydrates, protein, lipids, fiber, water, and minerals such as Calcium (Ca), Phosphor (P), Potassium (K), Magnesium (Mg), Sodium (Na), Sulphur (S), Aluminum (Al), Boron (B), Barium (Ba), Cobalt (Co), Chromium (Cr), Copper (Cu), Iron (Fe), Gallium (Ga), Lithium (Li), Manganese (Mn), Nickel (Ni), Lead (Pb), Strontium (Sr), and Zinc (Zn), as well as non-nutritional factors such as vicin and conovisin, biological and therapeutic factors (Prabhu and Rajeswari, 2018).

Peptides present in this plant have antioxidant, anti-inflammatory, free radical scavenging, and antibacterial effects (Karkouch et al., 2017). The flavonoid components of this plant inhibit the enzyme tyrosinase, which may be effective for cosmetic purposes such as whitening the skin, melasma, freckles, and age spots due to ageing (Allam et al., 2018; Karkouch et al., 2017). Furthermore, its phenolic components have antioxidant properties that play a role in liver and kidney diseases and diabetes by lowering their complications caused by oxidative stress (Mejri et al., 2018). This plant is a rich source of levodopa, which is converted to dopamine in the body and has been shown to improve the symptoms of Parkinson's disease (Abdel-Sattar et al., 2021; Singh et al., 2013). The effect of this plant may be attributed to the contraction of the dermal papillary vessels of the hair follicle due to the local effect of dopamine and, therefore, the weakening and thinning of the hair due to insufficient blood supply. Another cause can be that the vicin and conovisin in this plant are converted to aglycone and isouramyl by the effect of skin microflora (similar to the effect of the gastrointestinal microflora); these two substances may interfere with the production of testosterone in the skin and lead to its reduction., which will also be reduced as this plant reduces androgen-dependent hair (Koriem et al., 2021). According to the results of this study and if there are significant results in larger studies in the future, it may be possible to benefit from the effects of this plant for the treatment of hirsutism and hypertrichosis, as well as for cosmetic purposes such as removing unwanted hair.

## Materials and Methods

**Extract and cream and placebo preparation**

To prepare this product, the seeds and pods of the plant were first dried, then ground and mixed with 70% (v/v) ethanol. For every 100 g of bean powder, 300 ml of 70% ethanol was added and poured into a glass jar with a lid and then placed in a 37^o^C oven for 72 hr and shaken several times a day. These were then filtered using a Buchner funnel and Whitman filter paper. Rota evaporator was then used to concentrate the extract which was then mixed with 20% w/v with Farabi base cream (a base cream made by Farabi Co. Iran). To prepare the placebo, Farabi base cream was used and to change its color, 20 drops of medicinal brown dye were added for every 100 g of cream.

**Study design and interventions**

**Figure 1:**
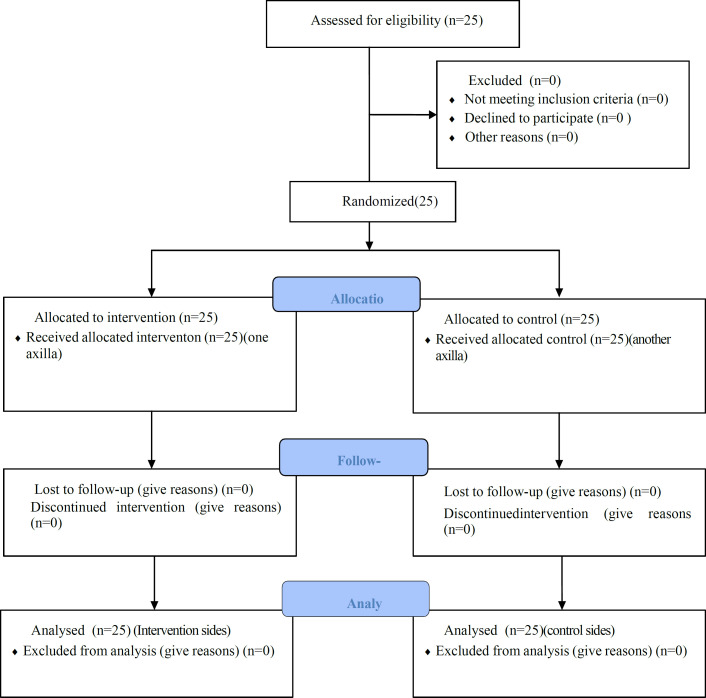
CONSORT 2010 flow diagram

In this study, a triple-blind clinical trial (performed for the first time), twenty-five female volunteers over the age of 15 years who were referred to the dermatology clinic of Imam Reza Hospital, Mashhad, Iran, were included to evaluate the effect of a cream containing 20% (w/v) broad bean extract on axillary hair (Figure 1). Inclusion criteria were being women over 15 years who wanted to reduce axillary hair with topical medication and did not undergo laser hair removal before and they consented to participate in this study. As exclusion criteria, all volunteers were examined, and those with any fungal or bacterial infection, dermatitis, scarring in the axilla, pregnancy or breastfeeding were excluded from the study. 

Furthermore, all volunteers were tested for glucose-6-phosphate dehydrogenase deficiency and were included only if it was negative. The volunteers were given a comprehensive description of the study and any possible complications before obtaining their consent. Patients were divided randomly via randomization.com site into two groups (A and B). A specific code was set for the right axilla of the intervention and the left axilla of the placebo, and another code was set for the right axilla of the placebo and the left axilla of the intervention. Two similar containers were prepared for each volunteer. The containers were placed in two packs (one intervention and one control). According to the code assigned to each pack, it was written on the containers for which axillary area (left or right) they were to be used. Each person was given two containers of cream per month.

These creams were applied to the underarm area with an approximate amount of one fingertip unit twice a day. Candidates were initially advised to use a small amount of both compounds on the inside of the arms and to wait for 48 hr, and only in the absence of irritation and allergic reactions to start application twice a day.

**Examinations**

Using a trichoscope, photographs of the axilla on both sides were taken in two views in order to count the number of hairs from a fixed distance of about 40 cm. It was done in the initial assessment before the study and then monthly. Photographs were also taken of the entire axillary cross-section on both sides. To check the average diameter of the hair, each axillary section on each side was divided into upper and lower halves, and by placing the tricoscope probe on both sides of the line, a cross-section with a diameter of about 1 cm on the skin was photographed. All hairs on both sections were measured in terms of the diameter; the average diameter of the hairs of the two sections was considered as a single number. Volunteers were advised to avoid shaving their hair three days before taking the photos and were urged to not using any other hair removal methods other than shaving during the study. The volunteers used both combinations for three months and were evaluated monthly.

At the beginning of the study, checklists including personal information, the number of times required to remove excess hair per week, and the type of method used previously to remove excess hair were completed.

At the end of the third month, a checklist containing a table where the participant could score the following was used: number of shaves per week compared to before the intervention (to assess the speed of hair growth), reduction in the number and thinning of hairs. Based on personal judgment, a maximum score of 10 for each of the above was given; a score of less than 3.3 was considered low, between 3.4 to 6.6 moderate and more than 6.7 significant. The scores were compared with the placebo on the opposite side. 

Volunteers were also evaluated for side effects during their visits. The information obtained from the volunteers was statistically analyzed, and the effectiveness of the broad bean extract was evaluated. In the final checklist, different types of side effects such as erythema, itching, burning, flaking, color and malodor of clothes and the option of other side effects were written, and the volunteers identified the type of complications they had experienced.

**Ethical statements**

This research study was approved by the Ethics Committee of Mashhad University of Medical Science (Code: IR.MUMS.MEDICAL.REC.1399.023) and the Iranian Registry of Clinical Trials (IRCT20200411047020N1).

**Sample size calculation**

This study is the first randomized clinical trial (blinded for volunteer, evaluator and analyst) to investigate the effect of this drug on body hair. The sample size calculation was conducted using G power software with regard to alpha 0.05 and beta 0.2. Considering the placebo effect as 5% and the expected and desired improvement reported by the researcher's consensus as 45% (Which was measured according to the thickness and number of hairs in the area), the sample size of 24 samples per group was calculated (the intervention group means the side where the drug was used and the control group means the side where the placebo was used(.

**Statistical analysis**

Data analysis was performed using SPSS software version 26, and a significance level lower than 0.05 was considered. Central and dispersion indices were used to describe the data. The Mac-Nemar test was used to describe the qualitative variables in pairs, the Wilcoxon test was used for the ranking variables, and the paired t-test was used to examine the quantitative variables. A linear mixed model was used to investigate the changes in quantitative variables between the two groups.

## Results

**Demographics**

This study was a triple-blind randomized clinical trial on 25 women with a mean age of 35.8±8.9 years (ranging from 16 to 50 years). Based on the paired t-test (Table 1), the difference between the intervention and placebo groups was insignificant in any of the three evaluations of their hair thickness. 

**The effects of the intervention on hair thickness**

Changes in hair thickness over time were examined using a linear mixed model. The reduction in hair thickness was significant over time (p=0.012, Table 1), but an insignificant difference in changes between the intervention and placebo groups was observed (p=0.595, Table 1).

**The effects of the intervention on hair number**

Paired t-test was used to evaluate changes in hair number (Table 2). The decrease in the number of hairs in the first month between the intervention and placebo group was insignificant despite a decreasing trend (p=0.164 and p=0.538, Table 2). However, in the second month, the number of hairs on the intervention side was significantly less than that on the placebo side (p=0.034, Table 2). In the third month, although the number of hairs in the intervention group was less than in the placebo, the difference between the two groups was not significant (p=0.073). A linear mixed model was used to investigate the general trend of changes in the number of hairs over time, showing no significant results (p=0.142, Table 2). 

Furthermore, there was no significant difference in the number of hairs between the two groups (p=0.401, Table 2).

**Table 1:** Comparison of hair thickness distribution (in millimeters) between the two groups by time

	**Intervention**	**Placebo**	p Value*
	Mean±Standard deviation Median (variation range)	Mean±Standard deviation Median (variation range)	
**Before Treatment**	0.02±0.083 (0.13–0.05) 0.08	0.02±0.085 (0.12–0.055) 0.09	0.595
**First month**	0.02±0.074 (0.1–0.035) 0.074	0.02±0.074 (0.12–0.014) 0.075	0.958
**Second month**	0.01±0.079 0.075 (0.055-0.1)	0.01±0.08 (0.105–0.055) 0.08	0.679
**Third month**	0.079±0.02 0.075 (0.055-0.14)	0.03±0.086 (0.150–0.055) 0.08	0.120

*Based on the paired t-test

**Table 2:** Comparison of hair numbers between the two groups by time

	I	P	p V*
	M±S	M±S	
	35.4±126.8 (47-175) 131	49.2±138.3 (243- 56) 123	0.164
	53.6±126.0 (257- 37) 120	59.9±129.8 (314- 49) 113	0.538
	49.5±119.2 (236- 38) 112	64.1±138.8 (286- 46) 125	0.034
	46.0±113.6 (209- 44) 110	52.0±128.6 (236- 49) 126	0.073

*Based on the paired t-test

**The effects of the intervention on the personal judgment of the volunteers on hair growth retardation**

The Wilcoxon test was used to compare the two groups in assessing the personal judgment of the volunteers. In terms of hair growth retardation (Table 3A), the number of participants who thought that their hair growth retardation was moderate and significant was more on the intervention side than the placebo side. However, the difference between the two groups was not significant in terms of individual judgment in slowing hair growth (p=0.119, Table 3A)

**The effects of the intervention on the personal judgment of the volunteers on hair number**

In terms of reducing the number of hairs, based on personal judgment (Table 3B), the percentage of people who thought that the reduction in the number of hairs was mild was 36% on the intervention side and 68% on the placebo side. On the other hand, the number of people who thought that reducing the number of hairs was moderate and significant was 32% on the intervention side and 16% on the placebo side. The difference between the two creams was significant in terms of personal judgment in hair number reduction (p=0.012, Table 3B). 

**The effects of the intervention on the personal judgment of the volunteers on hair thickness and lightness**

The difference between the two groups in terms of thinning and lightening of hair was found statistically significant based on personal judgment (Table 3C, p=0.020); This difference was significant in that on the intervention side, the percentage of people who thought that they had moderate hair reduction and significant thinning was higher than the placebo side. Twenty-four and twenty-eight percent of the subjects on the intervention side thought that thinning hair was moderate and significant, respectively. These percentages on the placebo side were 16% and 12%, respectively. A significant percentage of people (72.0%) believed that thinning and lightening of hair on the placebo side was mild (Table 3C).

**Complication**

Mac-Nemar test evaluated possible adverse effects (Table 4). The adverse effects were mild, and all reported adverse effects were related to the third month of drug use. Four people mentioned itching and erythema, three people said burning, two people mentioned scaling, and 96% of the participants mentioned staining of clothes and malodor on the intervention side (Table 4). But the difference between the two groups was significant only in terms of color and malodor of clothing (p<0.001, Table 4). 

**Table 3:** Comparison of the frequency distribution of hair (a) growth retardation, (b) reducing the number of hairs, (c) thinning and lightening of hair after treatment based on self-judgment between the two groups studied

		**Intervention**	**Placebo**	p-Value*
		**Frequency (Percent)**	**(** **Percent** **) ** **Frequency**	
	**Mild**	(52.0) 13	(64.0) 16	0.119
A	**Moderate**	(24.0) 6	(24.0) 6	
	**Significant**	(24.0) 6	(12.0) 3	
	**Mild**	(36.0) 9	(68.0) 17	0.012
B	**Moderate**	(32.0) 8	(16.0) 4	
	**Significant**	(32.0) 8	(16.0) 4	
	**Mild**	(48.0) 12	(72.0) 18	0.02
C	**Moderate**	(24.0) 6	(16.0) 4	
	**Significant**	(28.0) 7	(12.0) 3	

*Based on the Wilcoxon test

**Table 4:** Comparison of the frequency distribution of complications between the two groups

	**Intervention**	**Placebo**	p-Value*
	**Frequency (Percent)**	**Frequency (Percent)**	
**Pruritus**	(16.0) 4	0	0.125
**Erythema**	(16.0) 4	0	0.125
**Burning**	(12.0) 3	0	0.250
**Flake**	(8.0) 2	0	0.500
**Color and malodor of clothing**	(96.0) 24	0	0.001>

* Based on the Mac–Nemar test

## Discussion

Topical creams have many advantages, and they are easy to apply, time-effective, can be self-applied, and usually have few side effects. This has led them to be more popular than other methods. When effective, they can be used to remove physiological body hair and as an alternative treatment for pathological hairs such as hirsutism and hypertrichosis.

So far, no study has investigated the topical effect of bean-containing compounds on body hair reduction, with this study being the first case in this regard. 

In the books of traditional Iranian medicine, including Avicenna's Canon of Medicine, the effect of the broad bean on body hair removal has been mentioned (Sina, 1991). This plant is a rich source of levodopa, viscin and conovisin. 

The effect of this plant may be attributed to the contraction of the dermal papillary vessels of the hair follicle due to the local effect of dopamine and, therefore, the weakening and thinning of the hair due to insufficient blood supply (Shome et al., 2011; Singh et al., 2013; Stüttgen and Dreesen, 1979). Another cause can be that vicin and conovisin in this plant are converted to aglycone and isouramyl by the effect of skin microflora (similar to the effect of the gastrointestinal microflora); these two substances may interfere with the production of testosterone in the skin and lead to its reduction, which will also be reduced as this plant reduces androgen-dependent hair (Koriem et al., 2021). 

Various studies have examined the effects of herbal compounds such as fennel, *Curcuma aeruginosa*, and other topical drugs such as eflornithine and finasteride on hair reduction. Most cases are performed on facial hair due to idiopathic hirsutism, and only one study has examined the effect of *Curcuma aeruginosa* on axillary hair reduction, with positive effects being reported (Akha et al., 2014; Hamzavi et al., 2007; Javidnia et al., 2003; Srivilai et al., 2017; Tahvilian et al., 2015). In order to investigate the effect of *Curcuma aeruginosa* on reducing the density and diameter of hairs in the axilla, a type of camera was used which, via connecting to a computer, photographed an area of 1.2 x 1.3 cm in the area of the axilla and measured the number and diameter of hairs. Questionnaires were provided to patients to judge the effect of each drug and placebo on the axilla of both sides. Slower hair growth was reported in the photographic evaluation, but changes in the number of hairs on both sides were not significant (Srivilai et al., 2017). In our study, a trichoscope was used to assess the number and thickness of hairs; for personal evaluation, checklists were given to the volunteers who expressed their satisfaction with each axilla separately by scoring from zero to ten.

Although the present study is the first study on the effect of a topical bean composition on body hair reduction, its relative effect has been seen, such as reducing the number of hairs in the second month and the satisfaction of volunteers with its effect on reducing the number and thinning of hair. Perhaps using higher concentrations of bean extract, different formulations in the form of oils or lotions, using freshly crushed plant components instead of dried components, changing the solvent type, using it for a longer time (more than three months), may be associated with better results. Due to the fact that no significant side effects were seen in this study, if further evaluations prove its effectiveness, it may be used as a new treatment modality for body hair removal.

## Conflicts of interest

The authors have declared that there is no conflict of interest.
